# Determining the Quantitative Threshold of High-Frequency Oscillation Distribution to Delineate the Epileptogenic Zone by Automated Detection

**DOI:** 10.3389/fneur.2018.00889

**Published:** 2018-11-13

**Authors:** Chenxi Jiang, Xiaonan Li, Jiaqing Yan, Tao Yu, Xueyuan Wang, Zhiwei Ren, Donghong Li, Chang Liu, Wei Du, Xiaoxia Zhou, Yue Xing, Guoping Ren, Guojun Zhang, Xiaofeng Yang

**Affiliations:** ^1^Center of Epilepsy, Center for Brain Disorders Research, Capital Medical University, Beijing, China; ^2^Center of Epilepsy, Beijing Institute of Brain Disorders, Beijing, China; ^3^Neuroelectrophysiological Laboratory, Xuanwu Hospital, Capital Medical University, Beijing, China; ^4^College of Electrical and Control Engineering, North China University of Technology, Beijing, China; ^5^Department of Functional Neurosurgery, Xuanwu Hospital, Capital Medical University, Beijing, China; ^6^Department of Neurology, Beijing Tiantan Hospital, Capital Medical University, Beijing, China

**Keywords:** HFO, automated detection, epilepsy, surgery, threshold

## Abstract

**Objective:** We proposed an improved automated high frequency oscillations (HFOs) detector that could not only be applied to various intracranial electrodes, but also automatically remove false HFOs caused by high-pass filtering. We proposed a continuous resection ratio of high order HFO channels and compared this ratio with each patient's post-surgical outcome, to determine the quantitative threshold of HFO distribution to delineate the epileptogenic zone (EZ).

**Methods:** We enrolled a total of 43 patients diagnosed with refractory epilepsy. The patients were used to optimize the parameters for SEEG electrodes, to test the algorithm for identifying false HFOs, and to calculate the continuous resection ratio of high order HFO channels. The ratio can be used to determine a quantitative threshold to locate the epileptogenic zone.

**Results:** Following optimization, the sensitivity, and specificity of our detector were 66.84 and 73.20% (ripples) and 69.76 and 66.13% (fast ripples, FRs), respectively. The sensitivity and specificity of our algorithm for removing false HFOs were 76.82 and 94.54% (ripples) and 72.55 and 94.87% (FRs), respectively. The median of the continuous resection ratio of high order HFO channels in patients with good surgical outcomes, was significantly higher than in patients with poor outcome, for both ripples and FRs (*P* < 0.05 ripples and *P* < 0.001 FRs).

**Conclusions:** Our automated detector has the advantage of not only applying to various intracranial electrodes but also removing false HFOs. Based on the continuous resection ratio of high order HFO channels, we can set the quantitative threshold for locating epileptogenic zones.

## Introduction

More than 30% of epilepsy patients eventually develop refractory epilepsy (1) for which resection of the epileptogenic zone (EZ) is an important treatment (2). Accurate localization of the EZ is therefore key in determining the surgical outcome (2). The EZ is the area of the brain that is necessary and sufficient for initiating seizures (3). Unfortunately, so far there are no methods which can accurately locate the EZ. Over the past two decades, numerous studies have shown that the removal of areas showing high rates of high frequency oscillations (HFOs) is associated with a good-surgical outcome (4–8). Therefore, HFOs are considered a promising biomarker of the seizure onset zone (SOZ) or epileptogenic zone (4, 9, 10). However, none of these studies have applied the quantitative threshold of HFO distribution to delineate the EZ. Therefore, it is crucial to explore the quantitative threshold of HFO distribution to delineate the EZ.

HFOs are classified into ripples (80–200 Hz) and fast ripples (FRs, 200–500 Hz). Although visual analysis remains the gold standard for HFO analysis, this procedure is time consuming and subjective (4, 10). Therefore, developing an automated detector is critical (4, 11). Over the past two decades, several automated HFO detectors have been proposed, including the methods of Root Mean Square (RMS) (11), Short-time Linelength (12), Envelope (13), Radial basis function neural network (14), wavelet entropy (15), and others (16). However, most algorithms use the entire electroencephalogram (iEEG) segment to calculate the baseline. When the channel contains massive HFOs or high-frequency activities, the calculated baseline level will deviate significantly from the true baseline level, resulting in a significant drop in the accuracy of an automated detection. To solve this problem, Ren et al. proposed an algorithm that calculates the baseline by maximum distributed peak points, which worked well (10).

Recent studies showed that the stereotactic electroencephalogram (SEEG) has increasingly been used to locate the EZ (17), because the signal to noise ratio of signals recorded by different types of electrodes, is significantly different (18–20). In order to allow automated detectors to adapt to various intracranial electrodes, it is necessary to develop an automated detector that can be applied to a variety of electrodes.

When analyzing HFOs the original EEG signals need band-pass filtering (18). When using classical filtering methods, sharp transient events passing through a high-pass filter can result in “false” HFOs (Gibbs effect) (21, 22). It is necessary to remove these false HFOs (23, 24).

Here, we proposed an improved automated detector for HFOs. We first optimized parameters for SEEG and then added an algorithm to remove false HFOs in our detector. The automated detector and algorithm were created by our laboratory. Finally, we calculated the continuous resection ratio of high order HFO channels and compared this ratio with surgical outcomes, to determine a quantitative threshold of the HFO distribution to delineate the EZ.

## Materials and methods

### Patient selection

Forty-three patients with intractable epilepsy were enrolled between March 2016 and May 2017 from Xuanwu Hospital of Capital Medical University. All the patients had been implanted with intracranial electrodes (subdural or SEEG electrodes). The study was approved by the ethics committee of Xuanwu Hospital and all the patients signed the informed consent.

Three different datasets were acquired from these patients. The first dataset included 24 patients (48 channels, 2 channels/patients) implanted with the SEEG electrode and used to optimize parameters.

The second dataset consisted of 10 patients (2 channels/patient; 8 patients with SEEG electrodes and 2 patients with subdural electrodes) who were used to test our algorithm to identify false HFOs.

The third dataset included 26 patients (16 patients used SEEG electrodes and 10 patients used subdural electrodes) who met our inclusion criteria. These patients were used to study the relationship between the HFO distribution and surgical outcome to determine the quantitative threshold of HFO distribution to delineate the EZ. The patient inclusion criteria were as follows: (1) epileptic region had been surgically removed, (2) post-surgical follow-up for at least 12 months, and (3) the patient underwent post-surgical examination of magnetic resonance imaging (MRI). The criteria for exclusion were as follows: (1) the EZ was identified in bilateral hemispheres, (2) the EZ involved the occipital lobe, and (3) patients who experienced two or more surgeries. Patients' detailed information of third dataset is provided in Table S1.

### Electrode types and iEEG recording

The subdural electrodes (contact diameter of 4 mm with a 2.5 mm exposure, 10 mm spacing between contact centers) and SEEG electrodes with 8,10,12, and 16 contacts (0.8 mm diameter, 2 mm length, 1.5 mm between contacts; Beijing Huakehengsheng Healthcare Co., Ltd., Beijing, China) were implanted in the putative epileptogenic region based on previous non-invasive pre-surgical evaluation. The iEEGs were acquired using a 256-channel Nicolet recording system (Natus Medical Incorporated, San Carlos, CA, USA) with a sampling rate ≥2,000 Hz.

### Segment selection and visual analysis

In order to detect HFOs, we selected five minute segments during slow sleep period in which the delta band measured higher than 25% of all delta bands in a 30 second epoch. We also refer to the results of electrooculography and chin electromyography in determining the slow wave sleep period (10). All segments were selected from interictal periods, separated at least 2 h from seizures, and were transformed to a bipolar montage made of adjacent contacts.

Visual analysis was carried out by two reviewers. We used a zero-phase finite impulse response filter, and the cutoff frequencies were 80–200 Hz and 200–500 Hz for ripples and FRs, respectively. First, baseline segments were visually marked by one reviewer and considered as the negative gold standard (segments without high frequency activity, lasting for at least 200 ms). Then, HFOs were defined as the event where amplitude was clearly higher than the baseline with at least four consecutive oscillations. For each channel, the first minute of data was independently analyzed by two reviewers. The concordance between the reviewers was assessed using Cohen's kappa coefficient. If kappa > 0.5, the remaining 4 min of data were marked by one reviewer (10).

### Parameters optimization and automated detection of HFOs

In our previous study, parameter optimization was based on data from subdural electrodes (10). As SEEG has been increasingly used to locate the EZ in clinical practice and because the signal to noise ratio of an EEG recorded by different electrodes is significantly different, we considered it necessary to optimize the parameters for different electrodes. We randomly selected 48 channels from 24 patients implanted with SEEG electrodes, to optimize parameters using our previous method (10). In brief, we used the traversal method to repeatedly calculate sensitivity and specificity using different parameters and then computed the Youden index (sensitivity + specificity – 1) for different parameters. Parameters with higher specificity than sensitivity, as well as the highest Youden index, were regarded as the most suitable (25).

### The visual discrimination of false HFOs

**Figure 1:**
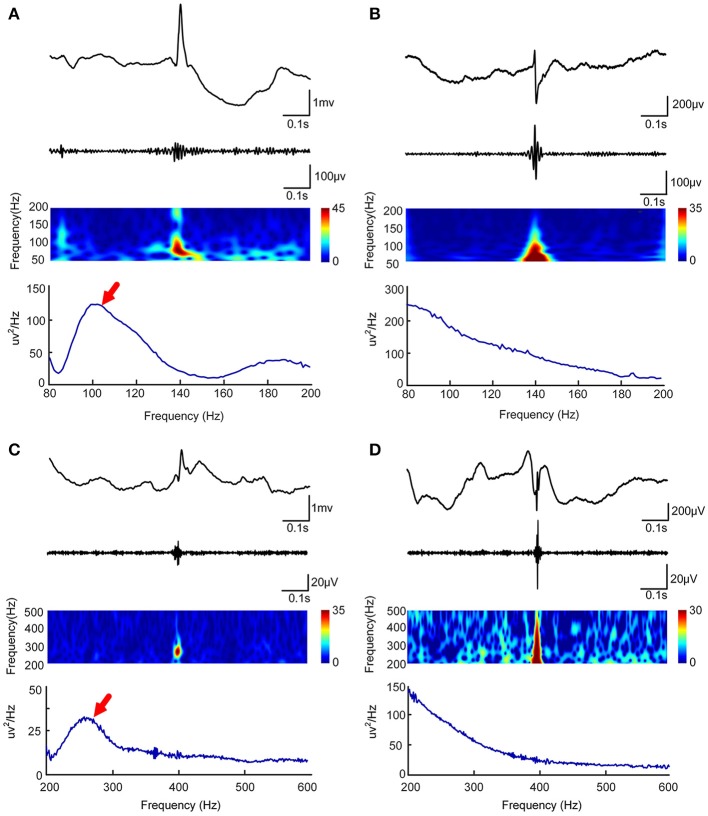
Visual and automated identification of false HFOs. In **(A–D)**, the first rows show one second of iEEG raw data, the second rows show the iEEG signal after the band-pass filtering [80–200 Hz for **(A,C)** and 200–500 Hz for **(B,D)**], the third rows show the Morlet wavelet spectrum, while the last rows represent a power spectral density map (PSD). The real HFOs appear as isolated islands **(A,C)** while false HFOs appear as a mountain-like shape **(B,D)** in the time-spectrum. On the power spectral density map, true HFOs have obvious peaks in the corresponding frequency range **(**arrows, **A,C)**, but these peaks do not appear in the false HFOs **(B,D)**.

To determine whether an HFO is caused by filtering, we used Morlet wavelet to prepare a time-frequency map to visually identify false HFOs. When an island-shaped high energy area on the time-frequency map at the corresponding time point of the HFOs was shown, the HFOs were marked as real HFOs. When a mountain-shape high energy area was shown, the HFOs were marked as false HFOs (22, 26, 27). We also plotted a chart of power spectral density (PSD) corresponding to the period of HFOs. If there was a significant power rise on the PSD, the HFO was considered true; otherwise, it was considered to be a false HFO. We visually identified true and false HFOs according to the two methods shown in Figure 1. Visual identification of false HFOs was processed by two reviewers. Concordance between the two reviewers was assessed using Cohen's kappa coefficient for each channel. When the kappa value < 0.5, the two reviewers jointly assessed the events until a consensus was established.

### Automated identification and removal of false HFOs

**Figure 2:**
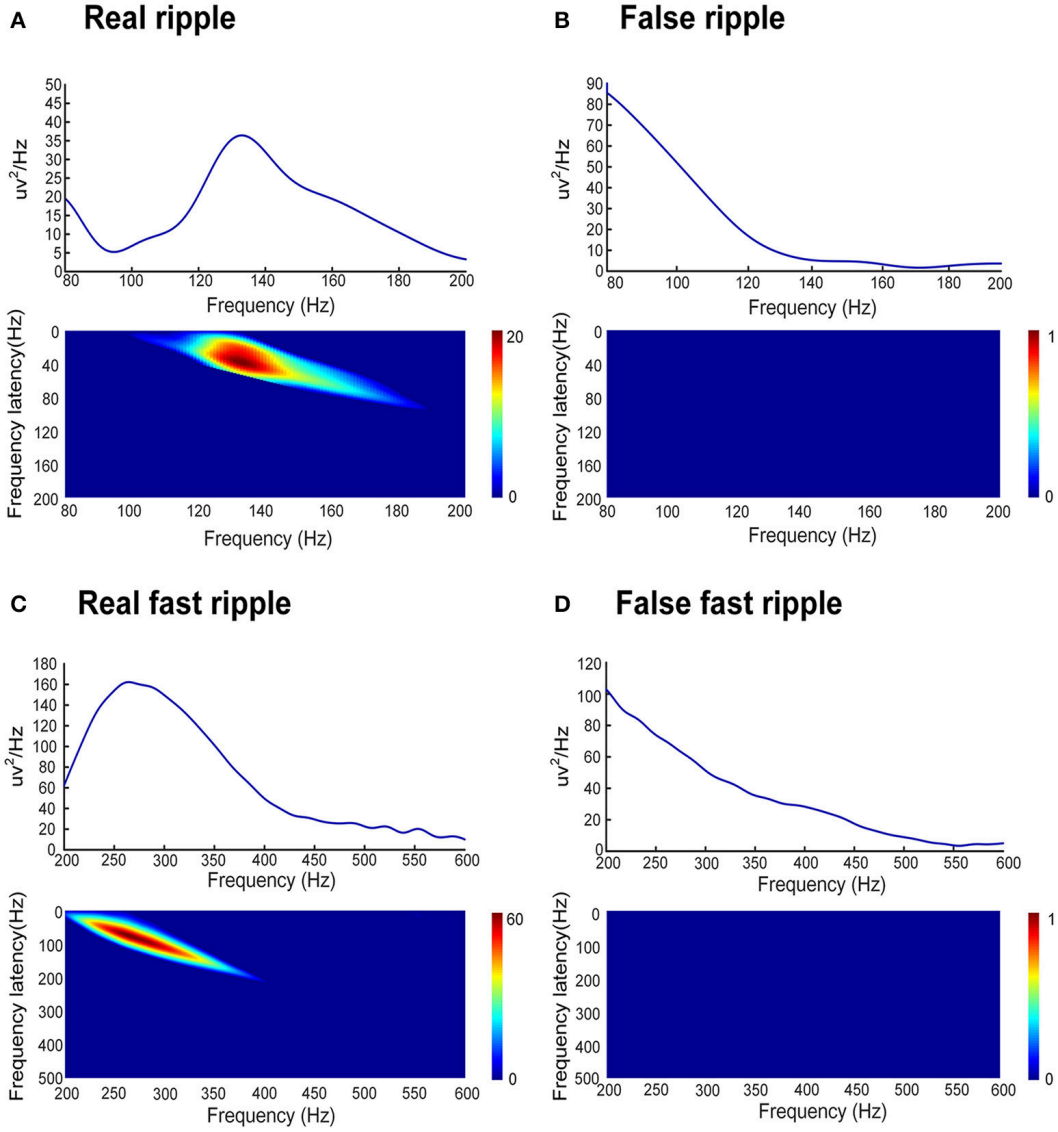
Automated identification of false HFOs. **(A,C)** represent true and false ripples; while **(B,D)** show true and false FRs, respectively. The top row shows the chart of power spectral density (PSD) corresponding to period of HFO. A true HFO will show a significant power rise on the PSD **(A,C)**, otherwise, the event should be classified as a false HFO **(B,D)**. The second row shows the accumulated power of frequency offset power difference of the phase space reconstruction where the HFO is located. The blue color shows that the accumulated power is lower than the baseline power; we count these instances as zero and classify them as a false HFO **(B,D)**. The red area shows the accumulated power. Instances where power is higher than the baseline power represent true HFOs **(A,C)**.

In order to automatically remove false HFOs, we proposed a novel algorithm to distinguish false HFOs from true HFOs. After plotting the PSD based on Morlet wavelets, the frequency offset power difference of phase space reconstruction was computed, at the middle time point of the corresponding HFO event. This power difference is calculated as Power(*f* )-Power(*f-*Δ*f*). When an HFO is false, it shows a trend of decreasing power as the frequency increases. In such instances, the frequency offset power difference is <0. Conversely, if the power of the frequency band where the HFO is located shows an increasing trend, the frequency offset power difference will then be >0. To identify complete information, we gradually increased Δ*f* values to reflect the characteristics of the frequency offset (our frequency offset value was set to 1–600). We then increased *f* from 80 to 600 Hz (in steps of 1 Hz) and increased Δ*f* from 1 to 200 Hz (ripples, in steps of 1 Hz) and 200 to 600 Hz (FR, in steps of 1 Hz). When the value of the frequency offset power difference was <0, the value was set to 0. Since the power of the baseline affects the detection of HFOs, the algorithm adds baseline power constraints. Since the power of HFOs was greater than the baseline power, if the value of the frequency offset power difference was less than the baseline mean power, it was also set to zero. Finally, we accumulated the frequency offset power difference of the frequency band where the HFO was located. If the value was 0, it indicated that there was no energy increase in the frequency bands was therefore regarded as a false HFO (Figure 2).

### Outcomes with respect to seizures

The post-surgical outcomes of patients were classified according to Engel's classification (28) as class I (free of disabling seizures), class II (rare disabling seizures), class III (worthwhile improvement), and class IV (no worthwhile improvement). We defined good post-surgical outcome as class I (seizure-free) and poor post-surgical outcome as class ≥ II (recurrent seizures).

### Determination of a quantitative threshold of HFO distribution to delineate the EZ

Twenty-six patients, who met our inclusion criteria, were used to study the relationship between the HFO distribution and surgical outcomes, in order to establish a quantitative threshold of HFO distribution, to delineate the EZ.

We automatically detected HFOs from all channels in the 26 patients using the optimized parameters. Next, we ranked all channels in a descending order according to the HFO rates for each patient. As mentioned before, we believe that the first channel that was not continuously removed from the highest ranking is the most important. We then calculated the ratio between the sum of channels, starting from the highest ranking to the first channel which was not continuously removed, and the total number of channels which detected HFOs. We referred to this ratio as a “Continuous resection ratio of high order HFO channels.”

$$\begin{array}{l} \text{Continuous resection ratio of high order HFO channels}\\ ={\text{ \#ChannelConRem}}_{\text{HFOs}}/{\text{\#Channel}}_{\text{HFOs}}\times 100\%, \end{array}$$

where #ChannelConRem_HFOs_ refers to the number of continuously removed channels from the highest ranking and where #Channel_HFOs_ represents the number of channels detected HFOs. Only those channels with HFO rates >1 event per minute were used for analysis (29). Removed channels were confirmed by comparing the fusion of pre-surgical MRI and CT and post-surgical MRI.

### Statistical analysis

Sensitivity and specificity were calculated as follows: sensitivity = true positive detections/visual marking; specificity = 1 – false positive/automated detections. Cohen's kappa coefficient was used to compare consistency between the two reviewers; a Kappa value < 0.5 implied poor consistency, while a Kappa value > 0.5 implied good consistency. Non-parametric Spearman's rank correlation was used to compare visual and automated analyzed results. The relationship between the continuous resection ratio of high order HFO channels with post-surgical outcome was analyzed with the Mann–Whitney U test.

All statistical analyses were performed using IBM SPSS Statistics 20 (IBM Corp., Armonk, NY, USA).

## Results

### Parameters optimization for sEEG signals

We visually marked the HFOs of the 5-min segment in the first dataset. A total of 3007 ripples and 1637 FRs were marked by two reviewers. In addition, we selected 3883 (ripples), and 2457 (FRs) baseline segments as the negative standard.

We used the traversal method to optimize the parameters. The optimized parameters for use with SEEG electrodes, were set as amplitudes, at which there were eight consecutive peaks higher than 3 SD above baseline mean amplitude, and six consecutive peaks higher than 10 SD above baseline mean amplitude (ripples); eight consecutive peaks higher than 3 SDs above the baseline mean amplitude and six peaks higher than 9.5 SDs above baseline mean amplitude (FRs).

The sensitivity and specificity of our automated detector, for patients who had SEEG electrodes implanted, were 66.84 and 73.2% (ripples) and 69.76 and 66.13% (FRs), respectively. Spearman's rank correlation between visually-marked and automated detection showed a correlation coefficient (r) of 0.962 for ripples and 0.884 for FRs.

### Automated removal of false HFOs

We visually analyzed the total HFOs and false HFOs from the second dataset. A total of 1874 ripples and 1208 FRs were acquired, and of these, 699 ripples were false ripples and 102 FRs were false FRs. Our algorithm then detected 568 false ripples and 78 false FRs. The sensitivity and specificity of our algorithm to identify false HFOs was 76.82 and 94.54% for ripples and 72.55 and 94.87% for false FRs.

**Figure 3:**
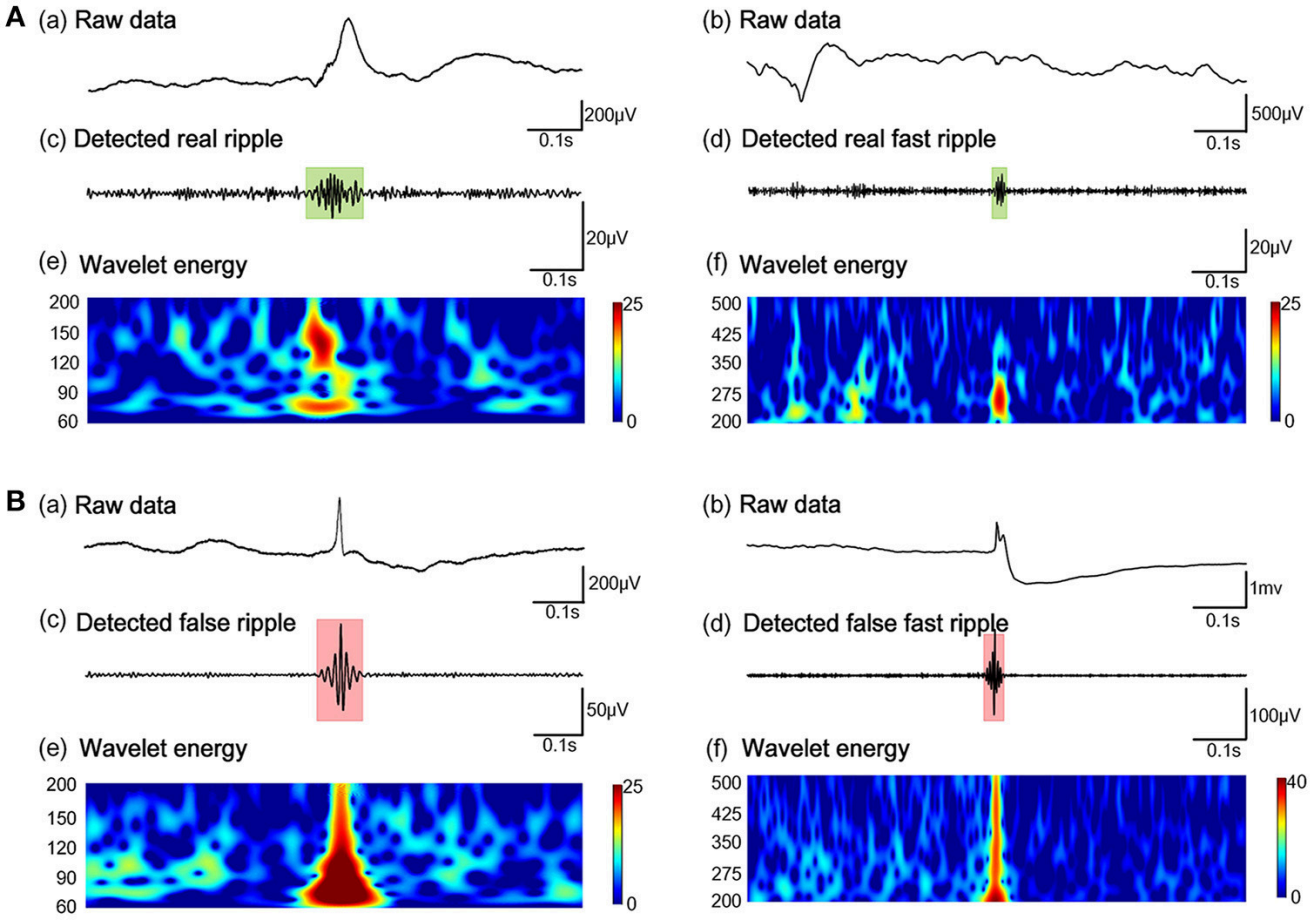
Example of the automated identification of true and false HFOs using our algorithm. **(A)** Real ripples and FRs. **(B)** Present false ripples and FRs. Panels **(Aa, Ab, Ba, Bb)** show one-second of raw iEEG signals. Panels **(Ac, Ad)** shows true HFOs were detected by our automated detector. Panels **(Bc, Bd)** show false HFOs which were identified by our automated detector. Panels **(Ae, Af, Be, Bf)** show the Morlet wavelet spectrum. The green regions and red regions represent the time points of HFOs.

Subsequently, our detector detected the 54854 ripples from 1340 channels and 30672 FRs from 495 channels, from 26 patients in dataset 3. Of these HFOs, our algorithm identified 11612 false ripples (21.17%) and 1501 false FRs (4.90%). Figure 3 shows the results from the automated identification of true and false HFOs, using our new algorithm. We found that the number of false ripples identified by our algorithm was much greater than that of the FRs, which may indicate that the ripple were more susceptible to the Gibbs effect.

We also compared the false HFO rate between SEEG and subdural electrodes (Table 1). In patients with SEEG electrodes, the median of detection rate of false ripples was 17.31% (Min–max: 4.20–42.89%) and 3.57% (Min–max: 0–75%) for false FRs. In patients with subdural electrodes, the median detection rate of false ripples and FRs was 29.83% (Min–max: 13.07–48.77%) and 10.37% (Min–max: 0–26.67%) for false FRs. The proportion of false ripples from subdural electrodes was significantly higher than that of the SEEG electrodes (*p* = 0.013), but not for the FRs (*p* = 0.096). These results implied that subdural electrodes may be more likely to cause false ripples after filtering (Table 1).

**Table 1:** Comparison of the rate of removing false HFOs of different electrode types.

	**SEEG**	**Subdural and depth**
Ripple (pre/post)	36151/29386	18703/13856
Fast ripple (pre/post)	22825/21833	7797/7338
Resection rate of ripple (Median, Min–max)	17.31 (4.20–42.89)	29.83 (13.07–40.74)*
Resection rate of fast ripple (Median, Min–max)	3.57 (0–75)	10.37 (0–26.67)

* *p = 0.013*.

### Continuous resection ratio of high order HFO channels

**Figure 4:**
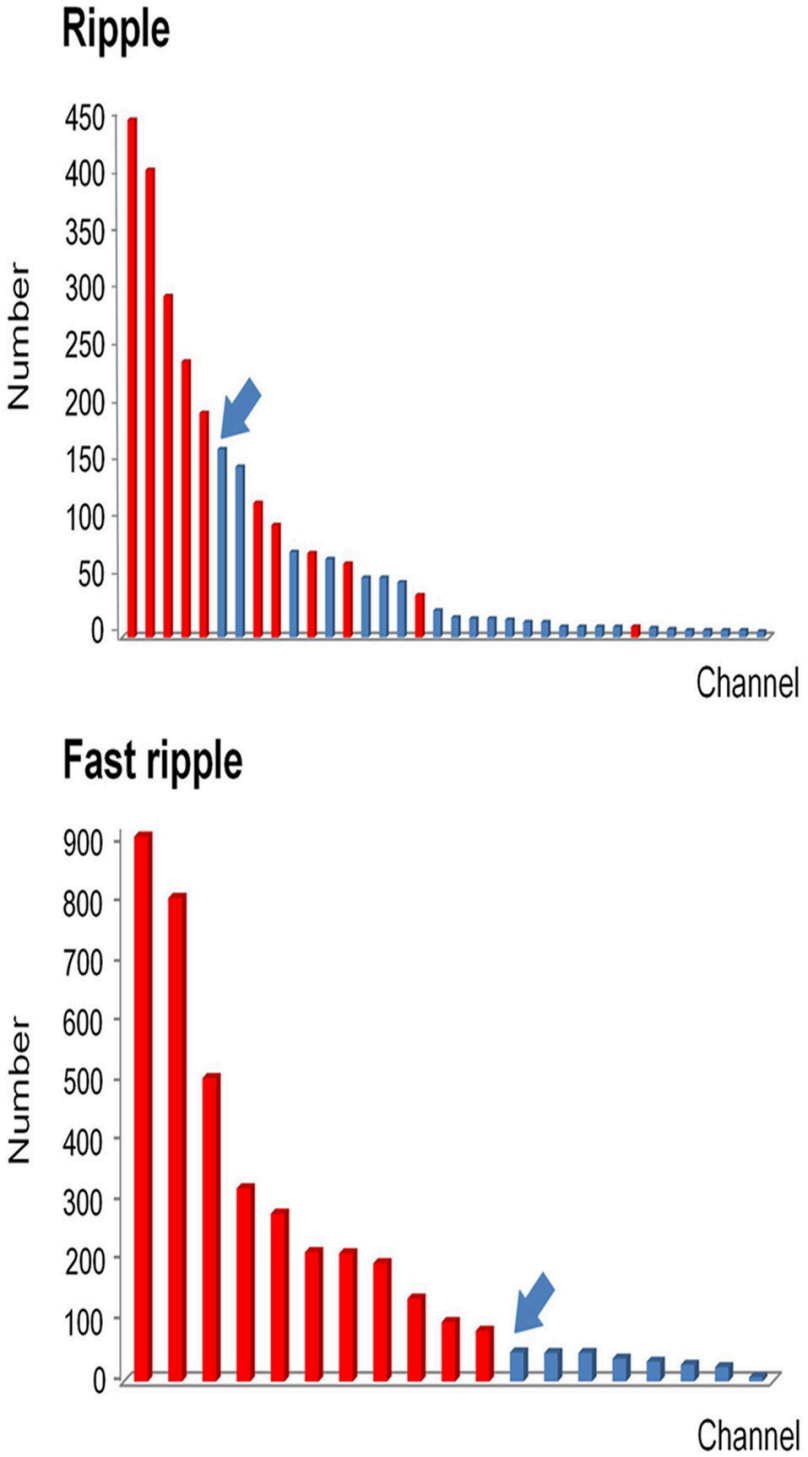
Example of how channels can be ranked in a descending order according to HFO rates. All channels which detected HFOs were ranked in a descending order according to HFO rates. Channels that were removed during the operation are marked in red, while non-removed channels are marked in blue. Then we identified the first channel that was not continuously removed from the highest ranking (arrows). Based on this channel, we then calculated the continuous resection ratio of high HFO order channels.

After removing false HFOs from the dataset 3, we finally detected 43242 true ripples and 29171 true FRs. We ranked the channels in descending order according to HFO rates for each patient. The number of channels consecutively removed from the highest ranking was then determined by a post-surgical MRI (Figure 4).

**Figure 5:**
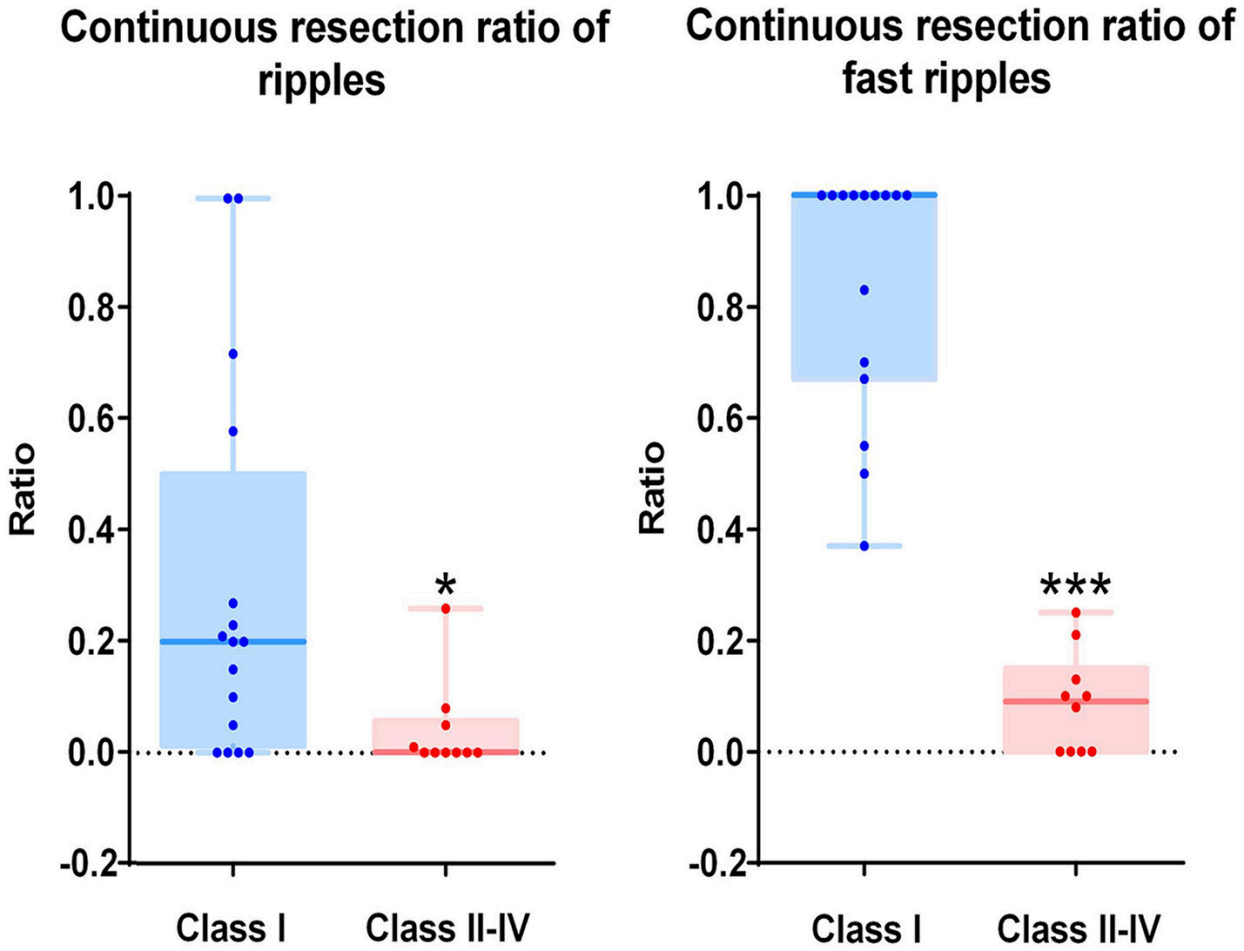
Correlation between the continuous resection ratio of high HFO order channels and post-surgical outcome. The continuous resection ratio of high HFO order channels exhibited statistically significant differences between the groups of patients with good outcomes and poor outcomes (*P* = 0.018 for ripples; *P* < 0.001 for FRs). No FRs were detected in patient 17. *P < 0.05; ***P < 0.001.

As mentioned above, we calculated the continuous resection ratio of high order HFO channels for each patient and compared the ratio with the patient's post-surgical outcome. For ripples, the median of the ratio in patients with a good post-surgical outcome was 0.2 (IQR: 0.0125–0.5025; 95% Confidence interval, CI 0.11 – 0.48; Min–max: 0–1), while the median of the ratio for patients with a poor post-surgical outcome was 0 (IQR: 0–0.0575; 95% CI – 0.02–0.1; Min–max: 0–0.26). In the FRs, the median of the ratio for patients with a good post-surgical outcome was 1 (IQR: 0.67–1; 95% CI 0.72–0.97; Min–max: 0.37–1) and for patients with a poor post-surgical outcome it was 0.09 (IQR: 0–0.15; 95% CI, 0.02–0.15; Min–max: 0–0.25). This ratio was statistically significant when comparing the groups of patients with good and poor outcomes (*P* = 0.018 in ripples; *P* < 0.001 in FRs Figure 5).

### Determination of a quantitative threshold of HFO distribution in order to delineate the EZ

**Figure 6:**
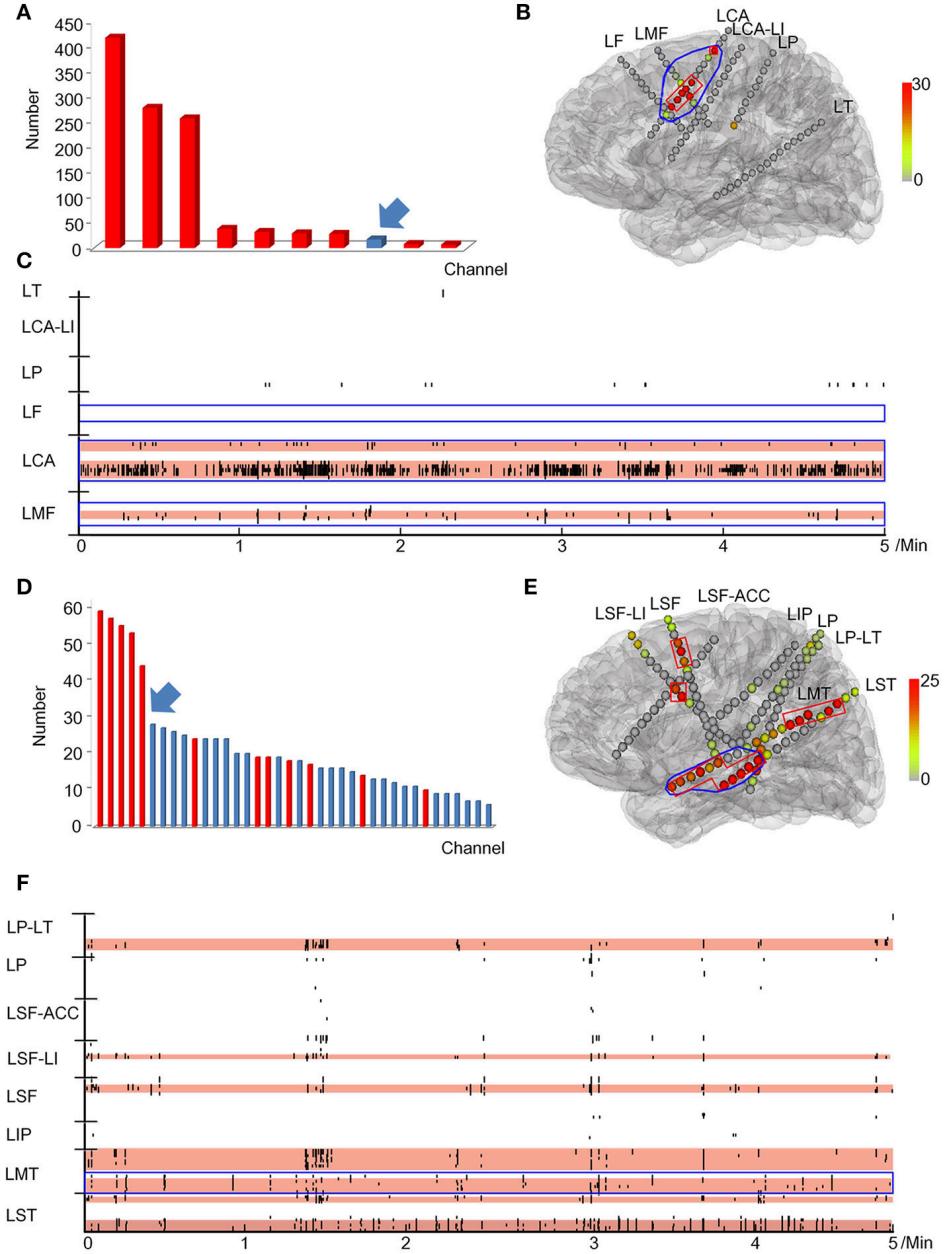
Example of a quantitative threshold for HFO distribution with which to delineate the EZ**. (A-C)** show the results of patient #13 (good post-surgical outcome) while **(D,E)** show the results of a poor post-surgical outcome. **(A,D)** show the rank in a descending order according to FRs rates. The channels which were resected are marked in red while the unresected channels are labeled in blue. Arrows indicate the first channel that was not continuously removed from the highest ranking. **(B,E)** show the FRs distribution on the individualized models of the patient brains. The number of HFO counts per electrode is represented by a different color. HFO counts are shown in red while the gray color indicates instances with a reduced HFO count. The resected area is delineated by the blue line and the EZ confirmed by our quantitative threshold is surrounded by a red line. LF, Left frontal; LMF, Left middle frontal; LCA, Left central area; LI, Left insula; LP, Left parietal; LT, Left temporal; LST, Left superior frontal; LMT, Left middle temporal; LIP, Left inferior parietal; LSF, Left superior frontal. **(C,F)** show the timing of all FRs detected in each channel by our automatic detector during a 5-min iEEG segment. The time and location of each FR are presented in terms of points. The resected area is delineated by the blue line. The pink regions show the EZ confirmed by our quantified threshold. In the patients with a good surgical outcome, the EZ was completely removed **(B,C)**. However, the EZ of patients with poor outcome was not completely removed **(E,F)**.

The relationship between the continuous resection ratio of high FRs order channels and post-surgical outcome, showed that the lowest value of the 95% confidence interval of this ratio, is 72% in patients with a good outcome. Therefore, we believe that when surgical resection reaches this threshold, the patient should have a good surgical outcome. Based on our results, we set a quantitative threshold of HFO distribution to locate the EZ at which the continuous resection ratio of high FRs order channels must be >72%. Figure 6 shows the channels ranked in descending order, according to HFO rates from two patients with a good outcome and a poor outcome, respectively. We also showed the relationship between the surgical resection regions and the channel distribution of FRs on patients' individual brain models. On these models, we showed the channels that generate FRs and highlight quantitative threshold for delineating the epileptogenic zone, according to the ratio. It is clear from our data, that the surgically-resectioned regions of patients with a good post-surgical outcome, completely covered the epileptogenic zone which was determined by the ratio. In contrast, in patients with a poor outcome, the epileptogenic zone that we identified, was completely removed during surgery.

## Discussion

In this study, we optimized the parameters for SEEG and added an algorithm to eliminate false HFOs to our detector. We then proposed a new concept, that uses the continuous resection rate of the high order HFO channel as a quantitative threshold, to delineate EZs.

### Optimizing parameters to improve the adaptability of our automatic detector

The inevitable subjective bias of visual analysis will lead to gaps between study groups, which seriously hampers the use of HFOs as clinical biomarker (4, 30). Therefore, over the past two decades, a variety of automated HFO detectors have been proposed. However, since most of these automated detectors used entire EEG segments to calculate the baseline (11–13, 16, 31, 32), baseline calculations in channels with many HFOs are not precise enough. To solve this problem, we proposed an automated detector, which uses the maximum distributed peak points method to calculate the baseline which can fit different HFO active states, thereby significantly improving detection accuracy (10). However, our previous parameters were optimized for subdural electrodes. Recently, the SEEG has been increasingly used to locate the EZ which represents a problem because differences in electrode implantation sites, the size and impedance, lead to differences in the signals recorded (18–20). In this experiment, we used the signals of SEEG to optimize the parameters for our automated detector, so that our automatic detector could adapt to various intracranial electrodes and detect HFOs accurately.

### Development of an algorithm to distinguish between true and false HFOs

The EEG signals need to be filtered before visual or automated analysis of HFOs (18). However, when using classical filtering methods, sharp transient events can result in “false” HFOs (21, 22). When studying the mechanisms of HFOs, or the relationship between HFOs and the epileptogenic zone, these false HFOs must be removed to ensure specificity of HFO detection. Waldman et al. developed a topographical method to distinguish true ripples from false ripples on epileptiform spike events arising from filtering (23). However, this method cannot identify false FRs. Numerous studies have demonstrated that FRs are significantly more meaningful than ripples, in locating the epileptogenic zone (7, 33–35). Therefore, identifying all false HFOs, especially false FRs, is very important for clinical application. Amiri et al. reported a method to identify false HFOs, arising from the filtering effect, by detecting oscillations in the raw signal at the time of sharp events (27). The authors mentioned that their method did not apply to scalp EEG. The detection of HFOs will eventually be extended to non-invasive methods, especially using scalp EEG signals. Therefore, the ideal HFO automated detector would be suitable for various EEG signals.

We proposed an algorithm to identify false HFOs by computing the frequency offset power difference of phase space reconstruction. Compared to others, our algorithm is not only applicable to various EEG signals but can also analyze ripples and FRs bands at the same time.

### Quantifying the threshold of HFO distribution to delineate the EZ

Numerous studies have demonstrated that higher rates of HFOs were observed within the SOZ or EZs (5, 8, 10, 36–40). The removal of areas generating high rates of HFOs is associated with good surgical outcomes (5, 7, 8, 10, 35, 41). Unfortunately, few studies have reported the use of the quantitative threshold of HFO distribution to delineate the EZ. Recently, Quitadamo and his colleagues reported EPINETLAB, an automated analysis software that can help researchers and clinicians to detect HFOs and identify the SOZ using iEEG/MEG data (42). They used this software to perform a preliminary validation analysis of EEG data in the ripple frequency band (80–250 Hz), from six patients with drug-resistant epilepsy who underwent pre-surgical evaluation with stereo-EEG (SEEG). Based on preoperative evaluation results, they reported that the algorithm could localize the SOZ with an average sensitivity of 81.94% and specificity of 96.03% with a reduction in computational load of more than 66% (43). However, this study only analyzed ripple frequency bands due to the limited sampling rate. A number of studies have found that fast ripples are more localized to the epileptogenic zone than ripples, therefore it is also important to validate the method on fast-ripple HFOs in the 250–500 Hz range.

In order to set a quantitative threshold of HFO distribution to delineate EZs, we ranked the channels in a descending order according to the HFO rates of each patient, separately for both ripples and FRs. Based on the theory of the EZ (3) and the fact that HFOs are mainly distributed in the EZ (7, 8, 10), we believe that the first channel which is not consecutively removed from the highest ranking is extremely important. If the channel is located in the EZ, then the post-surgical outcome will not be seizure-free (Class ≥II). Conversely, if the channel is located outside of the EZ, then the post-surgical outcome should be seizure-free (Class I). Based on this hypothesis, we proposed a new concept of the continuous resection ratio of high order HFO channels. The ratio is based on the proportion of continuously removed channels from high ranking HFOs, relative to the total number of channels with HFOs. Afterwards, we compared the relationship between this ratio and the post-surgical outcome of each patient. Our results showed that the ratio exhibited a highly significant difference between patients with a good and poor outcome in both ripple and FR bands. The ratio in the FRs to delineate the EZ was significantly better than in the ripples. Our results showed that the lowest value of the 95% confidence interval of the ratio for FR in a good post-surgical outcome group was at 72%. Therefore, we set the quantitative threshold for HFO distribution to delineate the EZ such as to continuously remove at least 72% channels from the highest ranking. To our knowledge, this is the first study to investigate a quantitative threshold based on HFO distribution to delineate the EZ.

### Distinction between physiological and pathological HFOs

The physiological HFO is another issue affecting the clinical application of HFOs, as physiological HFOs are completely independent of epileptic activity. Therefore, much attention has been paid on how to distinguish between physiological and pathological HFOs. A recent study that identified physiological HFOs in multiple regions of the brain suggested the need to assess interictal HFO activity relative to anatomically accurate normative standards, when using HFOs for pre-surgical planning (44). Nonoda et al. tried to distinguish epileptic HFOs from physiological HFOs, by detecting the relationship between HFOs and slow waves. They found that epileptic HFOs may be preferentially coupled with 3–4 Hz slow-wave, whereas physiological HFOs are more preferentially coupled with 0.5–1 Hz slow-wave during slow-wave sleep (45). Recently, Liu et al. proposed a method which used computer deep learning to automatically detect HFOs waveforms and was able to distinguish physiological and pathological HFOs based on waveform similarity (46). Although there have been several studies using a variety of techniques to distinguish physiological and pathological HFOs, it remains one of the key issues to be solved when applying HFO clinically.

In this study, we expanded the scope of application for our HFO automatic detector. We added an algorithm to remove false HFOs in our detector. We also proposed a concept for delineating the EZ, by setting a quantitative threshold according to the HFO distribution. Of course, this threshold was only set by our experimental data and needs to be validated with a large cohort of clinical data in the future. It is also necessary to develop methods to identify physiological and pathological HFOs and integrate these into our automatic detector, to promote HFO as a biomarker as early as possible, in the clinical diagnosis and treatment of epilepsy.

## Author contributions

XY, GZ, CJ, and XL contributed to the conception and design of this study. CJ, XL, and XY drafted the manuscript and prepared the figures. All authors contributed to the acquisition and analysis of data and to critical revisions of the manuscript.

### Conflict of interest statement

The authors declare that the research was conducted in the absence of any commercial or financial relationships that could be construed as a potential conflict of interest.
