# Supplementary material for: Determining the Quantitative Threshold of High-Frequency Oscillation Distribution to Delineate the Epileptogenic Zone by Automated Detection
Source: Front Neurol. 2018 Nov 13;9:889. doi: 10.3389/fneur.2018.00889 (PMC6243027; doi:10.3389/fneur.2018.00889)
Supplement: Supplementary file 1 [file Table_1.docx]

Table S1. Clinical information and electrodes implantation sites of patients in the third dataset

| Patient | Gender | Age/Age on set(y) | MRI | Electrode category | Implantation  sites | Removed  contacts | Engel classification |
| --- | --- | --- | --- | --- | --- | --- | --- |
| 1 | M | 20/3 | No lesion | Subdural/  depth | RSF, RMF, RIF, RP, RMF(d), RIF(d) | RMF 1-7,10-16; RSF 3-8,9-14;RIF5 10-13; RMF(d) 5-8; | III |
| 2 | F | 41/19 | No lesion | SEEG | RP-PCC; RP-PCC; PREC-MCC;RMF-MCC; RSPC-MCC; | PREC-MCC 3-6; RMF-MCC 1-4; RSPC-MCC 8-12; | I |
| 3 | M | 28/27 | HA(right) | SEEG | RP-RI; RP-RI(b); RIPL-RT;RMF-RMI; RO-RH; | RP-RI 2-3; RP-RI(b) 1-5;RIPL-RT 1-3,8-10 | III |
| 4 | M | 35/2 | RF | SEEG | LMF-lesion;PREC-lesion;LP-lesion(b);LP-lesion;PSTS-lesion; | PREC-lesion1-13;  PSTS-lesion8-13; LMF-lesion 1-13; | II |
| 5 | M | 17/10 | HS(right) | SEEG | RIF-RGO;RMF-RC;RMF-RSI;RMF-RMI; RT-RH;  RIT-RH | RT-RH 1-6;  RIT-RH 1-5; | I |
| 6 | F | 35/25 | HS(right) | Subdural/depth | RTP; LTP; RT; LT;  RH(d); LH(d); RI(d); | RTP 5-8; RT 5-8;  RH(d) 1-12; | I |
| 7 | M | 23/17 | GMH(LV) | SEEG | LP-LV;LP-LP;LMT-LH;LMT-MCC;  LIP-LT; LIF-ACC; | LMT-LH 1-16;  LIP-LT 1-7; | I |
| 8 | M | 24/18 | HS(left) | SEEG | LT-LH; LO-LH; LSF-LI; LIF-LMI;  RO-RH; RSF-RI; | LT-LH 1-9; LO-LH1-6;  LSF-LI 1-6; | IV |
| 9 | M | 10/7 | Encephalomalacia | Subdural | LSF(b); LMF(b); LIF(b);LT,; LTP; LPO; | LSF(b) 8,15-16;LMF(b) 7-8,15-16;LIF(b)7-8,15-16; LT6-8;LTP5-8,15-16; LPO6-8; | III |
| 10 | F | 12/8 | No lesion | SEEG | RT-RH; RIP-RI; RSP-RI;RP-PCC;RPREC-MCC;PREC-RI; RT-RMT; | RIP-RI 12-16; RSP-RI 12-16;RP-PCC 12-16; | III |
| 11 | M | 23/1 | RF | SEEG | RMF-lesion;RSF-lesion;RSF-RI;RIF-RMI; RO-RH;RMF-ACC; | RMF-lesion 1-16; RSF-lesion 1-16;  RSF-RI 10-16; | I |
| 12 | M | 5/1 | No lesion | Subdural/  depth | LF; LMF; LIF;  LCA; LSF(d); LSF(b,d);LPREC(d); | LSF(d) 1-8; LSF(b,d)1-4;  LPREC(d) 4-7; | I |
| 13 | F | 26/5 | RF,LF | SEEG | LMF-MCC;LCA-lesion;LF-MCC;LP-MCC; LCA-LI; LT-LH; | LMF-MCC 3-10; LF-MCC 4-8;  LCA-lesion 4-14; | I |
| 14 | M | 21/10 | No lesion | SEEG | LST-LH; LMT-LH; LIP-LI; LSF-ANT;  LSF-LI; LSF-ACC; LP-PCC; LP-LT; | LST-LH 1-6; LMT-LH 1-6; | II |
| 15 | M | 29/5 | HS(right) | SEEG | RST-RA; RI-RH; RP-PCC; RI-ACC;  RIF-RF; LO-LH; | RST-RA 1-6; RI-RH 1-5; RI-ACC 1-7; RIF-RFB 1-6; | I |
| 16 | F | 13/10 | LC | SEEG | LMF-lesion;LP-lesion;LCS-PARC;LMF(b)-lesion;PREC-lesion; | LMF-lesion 1-3;LP-lesion 1-8;LMF(b)-lesion1-16; PREC-lesion1-4; | I |
| 17 | M | 16/15 | RF | SEEG | RSF-MCC;RMF-ACC; RMF-PCC; RI-RA; RSF-lesion; LMF-ACC; | RSF-MCC7-11;RMF-ACC 13-16;RI-RA 10-16; RSF-lesion 1-10 | I |
| 18 | F | 21/9 | LI, LF | SEEG | LMF-LI;LCA-LMI;LMF-broca; LP-LI;LP-LT; LT-LH;LMF-LMI; | LCA-LMI 1-5; LP-LI 1-5; LP-LT1-9;LMF-LMI 1-4; | II |
| 19 | F | 13/12 | No lesion | SEEG | LCA-SMA;LMF(b)-lesion; LIF-LI; LMF-lesion; | LCA-SMA 1-4; LMF(b)-lesion 1-16;  LMF-lesion 1-16; | I |
| 20 | M | 19/14 | No lesion | SEEG | RP-RSI;RIP-RI;RT-RT(B); RO-RH; LO-LH; | RIP-RI1-8; RT-RT(B) 1-8; RO-RH 1-10; | II |
| 21 | M | 7/1 | LP, LT, LF, RP, PT | Subdural | LP; LT; RP; RTP; | LT 1-4,9-12;LP 1-4, 9-12,17-20,25-28; | III |
| 22 | F | 28/10 | SS | Subdural/depth | LF; LP; LT; LT(d); LI(d); | LF 6-8,14-16; LP 1-5,9-13;LT 1-4, 9-11; | I |
| 23 | M | 16/10 | HS(left) | Subdural | LT, LLF,LH | LLF 1-4,9-12; LT 17-22, 25-30；LH 1-4; | I |
| 24 | F | 42/2 | HS(right) | Subdural | LH, RH, RT | RT 1-6, 9-14; RH 1-8; | I |
| 25 | M | 24/1 | No lesion | Subdural/depth | LF, LIF, LT, LIT, LH | LIF 1-3, 9-11; LT 1-6, 9-14; LIT 1-5, 7-11; | I |
| 26 | F | 35/30 | No lesion | Subdural | LT, LPO, LH | LT 1-3, 7-12; LPO 5-8, 13-16,21-24, 30-32; LH 1-8; | I |

RF: Right frontal; RSF: Right superior frontal; RMF: Right middle frontal; RIF: Right inferior frontal; RP: Right parietal; RSP: Right superior parietal; RIP: Right inferior parietal; RI: Right insula; RSI: Right superior insula; RMI: Right middle insula; RT: Right temporal; RMT：Right middle temporal; RIT: Right inferior temporal; RTP: Right temporal pole/parietal; RTT: Right transverse temporal; RO: Right occipital; RH: Right hippocampus;RA: Right amygdala; RSPC: Right superior parietal cortex; RIPL: Right inferior parietal lobe; LSF: Left superior frontal; LMF: Left middle frontal; LIF: Left inferior frontal; LLF: Left lateral fissure; LP: Left parietal;LIP: Left inferior parietal; LI: Left insula; LMI: Left middle insula; LT: Left temporal; LST: Left superior temporal; LMT: Left middle temporal; LTP: Left temporal pole/parietal; LTT: Left transverse temporal; LO: Left occipital; LH: Left hippocampus; LPO: Left parietal occipital; PARC:Paracentrallobule;PREC: Precentralgyrus; PSTS: Postcentralgyrus; RGO: Right gyriorbitals; RC: Right cingulate; ACC: Anterior cingulate cortex; MCC: Middle cingulate cortex; PCC: posterior cingulate cortex; SMA:Supplementary motor area; LV: Lateral ventricle; LCA: Left central area; LCS: Left central sulcus; ANT: Anterior nucleus thalamus; HA: Hippocampus atrophy; HS: Hippocampus sclerosis; GMH:Graymatterheterotopia; SS: Sphenoid sinus; (b):back; (B):Base; (d):depth;
